# Current Advances and Future Perspectives of Advanced Polymer Processing for Bone and Tissue Engineering: Morphological Control and Applications

**DOI:** 10.3389/fbioe.2022.895766

**Published:** 2022-05-26

**Authors:** Tongrui Zhang, Min Nie, Yijun Li

**Affiliations:** State Key Laboratory of Polymer Materials Engineering, Polymer Research Institute of Sichuan University, Chengdu, China

**Keywords:** advanced polymer processing, micro extrusion, micro injection molding, 3D printing, bone repair, biomedical engineering

## Abstract

Advanced polymer processing has received extensive attention due to its unique control of complex force fields and customizability, and has been widely applied in various fields, especially in preparation of functional devices for bioengineering and biotechnology. This review aims to provide an overview of various advanced polymer processing techniques including rotation extrusion, electrospinning, micro injection molding, 3D printing and their recent progresses in the field of cell proliferation, bone repair, and artificial blood vessels. This review dose not only attempts to provide a comprehensive understanding of advanced polymer processing, but also aims to guide for design and fabrication of next-generation device for biomedical engineering.

## Introduction

With the development of polymer processing, functional polymer materials and devices for biomedical engineering have ushered in a huge development, and new concepts, new technologies and new methods are still being explored on a global scale (Abdolmaleki et al., 2021; Guo et al., 2021; Zhang et al., 2021; Chen et al., 2022). Among them, the ingenious combination of advanced polymer processing techniques and functional polymer composites demonstrate epoch-making significance of next-generation medical device such as cell proliferation (Motealleh et al., 2021), bone repair, (Xue et al., 2021), drug release, (Kim et al., 2021), artificial blood vessels (El-Ghazali et al., 2021) and thus have attracted extensive attention from academia and industry.

The performance of polymer materials and device is not only derived from the structural characteristics of the molecular chain, but also strongly depends on the morphology and structure formed by the effect of stress and temperature during processing (Ho et al., 2012; Zhang et al., 2019). Advanced polymer processing technology can generate a unique stress and temperature filed with the assistance of special processing equipment and thus enables polymer materials to exhibit excellent properties which are different from conventional processing, exemplified by the helical stress field formed in rotation extrusion (Pi et al., 2019; Sun et al., 2019), the ultra-high shear in micro-injection molding (Jiang et al., 2018; Zhao et al., 2022) and so on. These materials and device provide a broader toolbox for practical clinical application.

In this work, we reviewed the recent advanced processing technology stepping from clarifying the special stress and temperature field during processing and then demonstrated its current and potential applications in cell proliferation, bone repair engineering and artificial blood vessels. In addition, based on the outlined challenges and limitations of advanced processing technology, we summarized the prospects and development directions of advanced processing technology to provide feasible ideas for inspiring future work.

## Rotation Extrusion Technology

### Principle of Rotation Extrusion Technology

**FIGURE 1:**
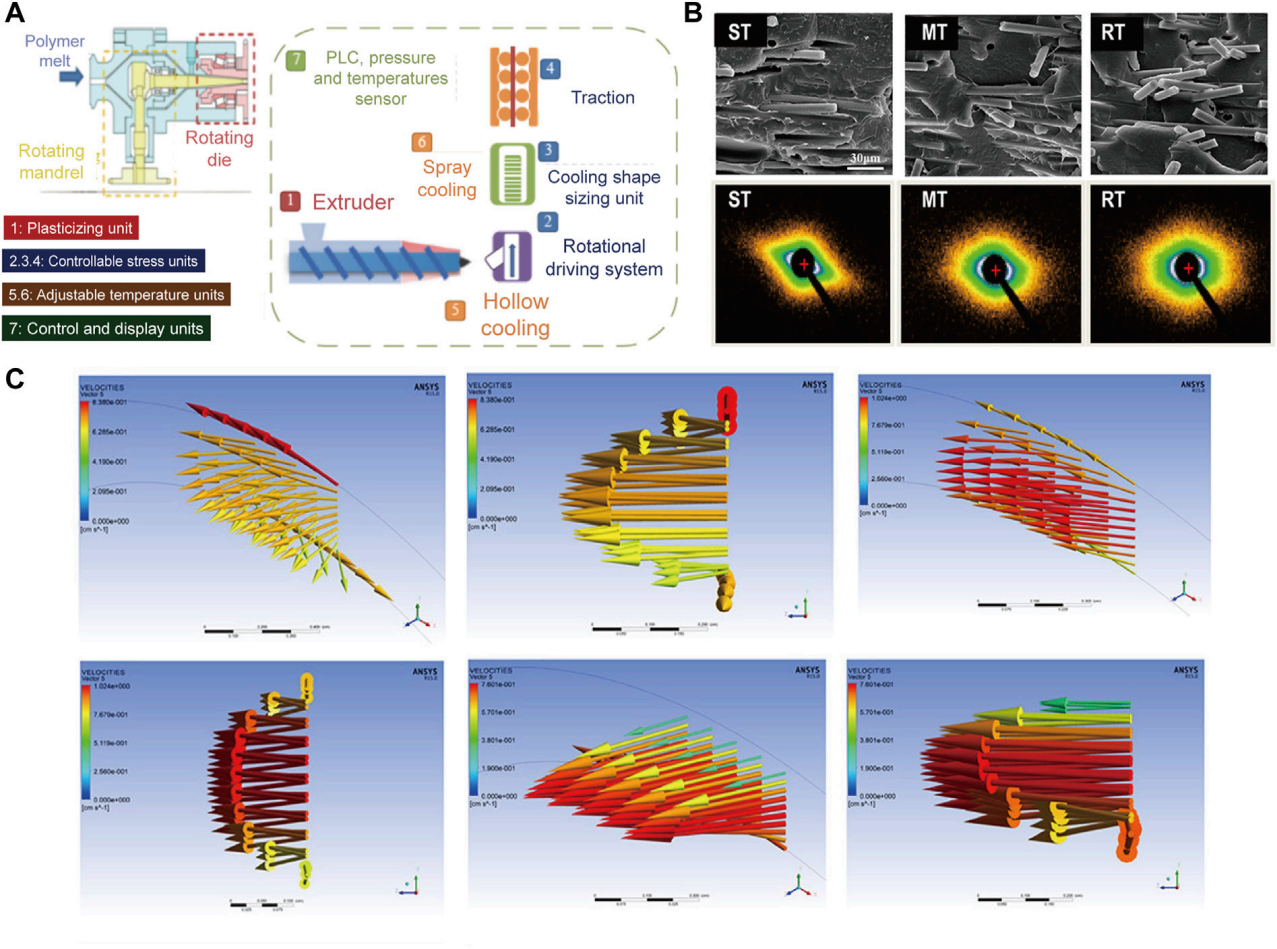
**(A) **Schematic diagram of rotation extrusion rheometer; **(B)** Scanning electron microscopy (SEM) and small-angle X-ray scattering (SAXS) results of the MT, ST, and RT tubes; **(C)** Flow characteristics of MT, ST, and RT obtained from Polyflow (Li et al., 2021b). Copyright 2021, American Chemical Society.

Differing from one-dimensional axial flow in conventional extrusion process (Lu et al., 2020), helical stress filed is generated in rotation extrusion through the controlled rotation of the die and the mandrel, which realizes the multi-dimensional controllable flow of the melt and improves the functionality of the material (Li Y. et al., 2019). Figure 1A shows the schematic illustration of the rotation extrusion rheometer (Li et al., 2021a). The rotation extrusion rheometer can accurately control and exert hoop stress during extrusion, and thus helical motion can be achieved in combination with extrusion and traction. At the same time, the cooling media with various type, pressure and temperature can be adjusted into the polymer tube through the hollow mandrel, so as to regulate the temperature gradient and solidify the off-axis reinforcement phase structure in the polymer tube. During rotation extrusion, fiber network with different helical feature can form (Figures 1B,C) (Li et al., 2021a) Specifically, unidirectional orientation off the extrusion direction can form during syntropic rotation extrusion (ST) and mandrel rotation extrusion (MT), while intersected network with a gradient layer forms in reverse rotation extrusion (RT). The former can induce the reinforcement structure to deviate from the axial orientation and thus to reinforce the hoop strength of the tube, while the latter can form functional network inside the tube and broaden the application. During rotation extrusion process, the additional hoop stress field exhibits great significance in regulating the crystal morphology of the polymer. The increased shear from hoop direction can promote the stretching of molecular chains to enhance crystallinity and simultaneously promote the formation of shish-kebab structure (Yang et al., 2019), in which the anisotropic nature endows materials with higher strength and modulus.

### Application of Rotation Extrusion Technology in Preparing Medical Tube

Medical catheters are the most important application direction of polymer tubes (Geyer et al., 2019), mainly including surgical catheters (Hu et al., 2018), treatment and nursing catheters (Neoh et al., 2017), and interventional medical catheters (Liu et al., 2021). Interventional therapy is a newly developed medical technology, where catheters and other interventional medical devices can enter the human body to reach the target lesion location and to achieve precise and efficient treatment effects (Jung et al., 2020; Hwang et al., 2022). However, due to the thin wall, the mechanical properties of polymer tubes are generally low. Tubes are prone to kinking and collapse under external loads, causing fatal damage inside the human body (Wang et al., 2020a). Therefore, enhancement of mechanical properties within a limited size is an important development direction of the polymer tube.

**FIGURE 2:**
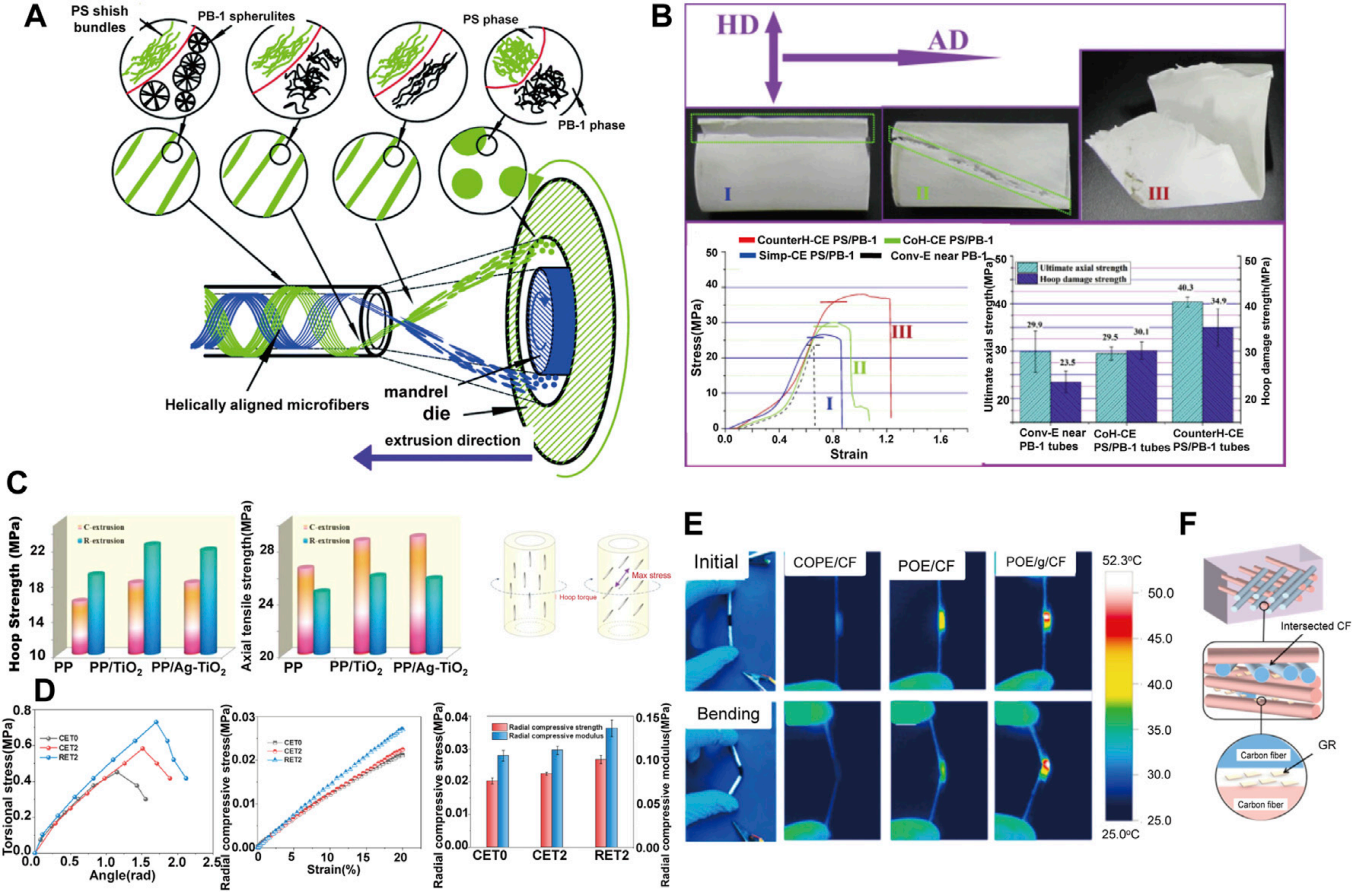
**(A)** Schematic diagram of *in situ* fibrillation by rotation extrusion technology (Liu et al., 2014) Copyright 2014, Elsevier; **(B)** Failed composite tubes with different microfiber’s configuration and the corresponding stress–strain data (Liu et al., 2015) Copyright 2015, Elsevier; **(C)** Hoop strength and axial strength of the tubes prepared at different conditions and stress analysis of hoop torque imposed on polymer tubes (Zhang K. et al., 2018) Copyright 2018, Elsevier; **(D)** Radial mechanical properties of POE/MMT tubes (Yang C. et al., 2021) Copyright 2021, Elsevier; **(E)** Infrared thermal images at the maximum temperature under voltage of 5 V with or without bending strain of various tubes. POE/CF referred to convention extruded tubes; **(F)** The schematic illustration of the confining effect of the GR on CF network (Li et al., 2021b) Copyright 2021, Elsevier.

The helical force field during rotation extrusion can induce the reinforcing fibers to deviate from the axial alignment, and parallel to the maximum stress in the torque deformation. This unique morphology can endows the microtube with excellent torsional strength to achieve annular strengthening (Nie, 2013; Sun et al., 2019). Liu(Liu et al., 2014) conducted a pioneering work in preparing medical tube with reinforced hoop strength *via* rotation extrusion, where polystyrene (PS) microfibers with high molecular orientation were helical aligned by the helical convergent flow during polybutene-1 (PB-1) tubing processing (Figure 2A). Moreover, the mechanical strength of the microtubules can be further improved by further controlling the rotation mode of the rotation extrusion. Liu (Liu et al., 2015) demonstrated that a reverse helical configuration of PS microfibers was constructed by forming a reverse helical convergent flow in reverse rotation extrusion, which effectively eliminating the relative slip between fibers. The prepared PS/PB-1 composite tube exhibits significantly enhanced mechanical properties in axial direction (40.3 PMa) and hoop direction (34.9 MPa), which are 34.8 and 48.5% higher than those prepared by conventional extrusion, respectively (Figure 2B).

Similarly, fillers with anisotropic nature are ideal candidate for rotation extrusion. Zhang and Li (Li X. et al., 2015; Zhang et al., 2018b; Pi et al., 2020) enhanced the circumferential strength of polypropylene (PP)/TiO_2_ tubes by rotation extrusion (Figure 2C). It is notable that these work proved the universality and customizability of the rotation extrusion technology, since by adjusting the type of filler to give the pipeline additional functionality. For instance, TiO_2_ can improve the antibacterial ability of the pipeline (Alotaibi et al., 2020). Yang (Yang C. et al., 2021) also fabricated reinforced polyolefin elastomer (POE)/montmorillonite (MMT) tubes by rotation extrusion. MMT formed a natural nacre-like structure under helical force field, which improves the hoop strength of the tube, and at the same time shows a strong ability to resist radial compression deformation (Figure 2D). The enhanced anti-deformation ability perfectly fits the needs of bone repair materials, establishing a foundation for the application in the field of bone repair. Moreover, reverse rotation can form a multi-axial ordered structure in polymer melt, which is beneficial to reduce the conductive percolation, form a conductive network, and prepare a new type of polymer functional microtubules. For example, a flexible tubular electrode is prepared by using the principle of Joule heating, which has wide application prospects in hyperthermia under complex conditions such as trachea and intestinal tract (Figures 2E,F) (Li et al., 2021c).

## Electrospinning

### Principle of Electrospinning

**FIGURE 3:**
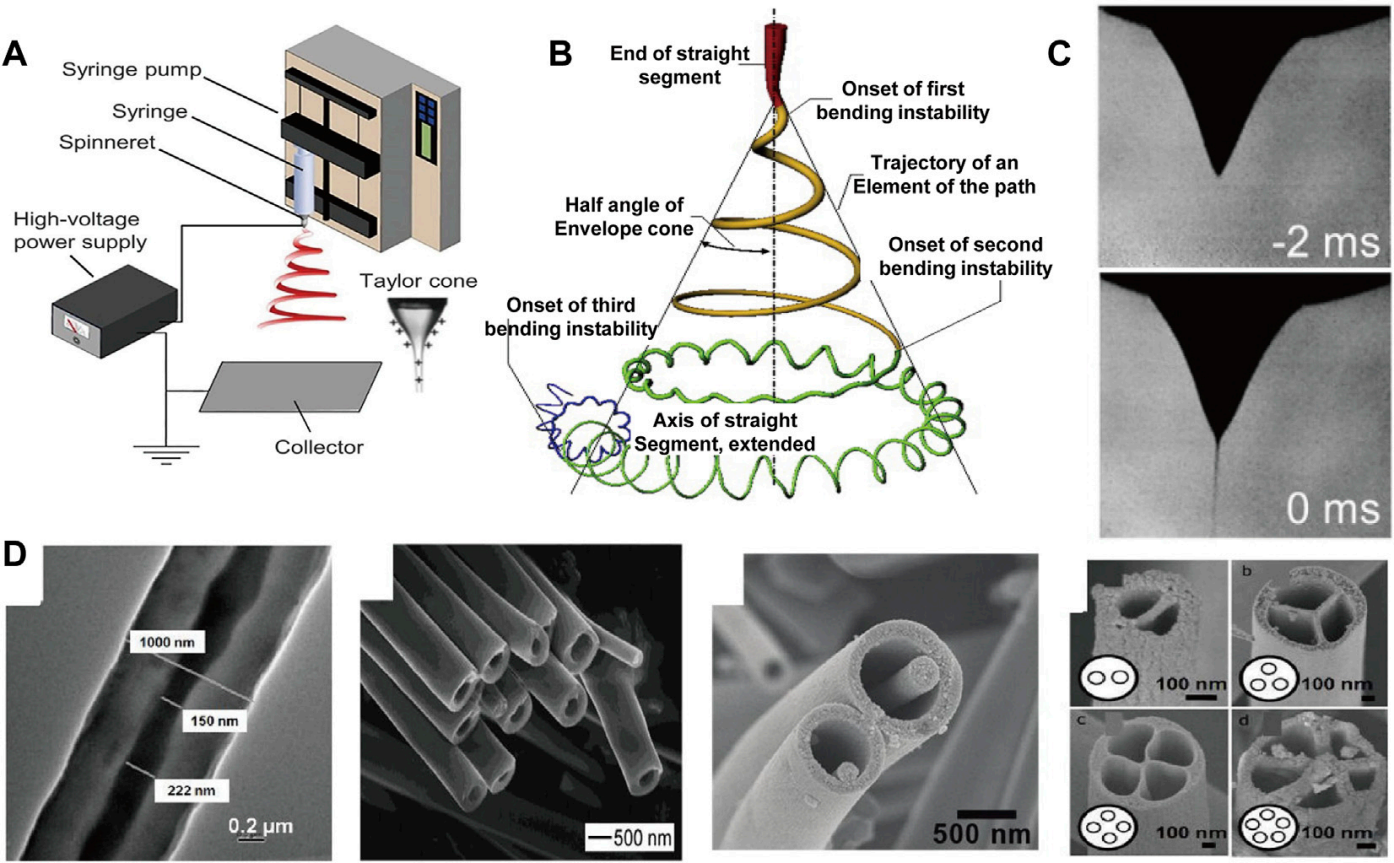
**(A)** Basic setup for electrospinning (Xue et al., 2019) Copyright 2019, American Chemical Society; **(B)** Schematic diagram of the prototype instantaneous position of the electrospinning jet path (Xue et al., 2019) Copyright 2019, American Chemical Society; **(C)** Reprinted with permission from ref (Xue et al., 2019) Copyright 2019, American Chemical Society; **(D)** Structural diversity of the individual electrospinning fiber (Sun et al., 2003; Li and Xia, 2004b; Zhao et al., 2007; Chen et al., 2010) Copyright 2004, American Chemical Society Copyright 2007, American Chemical Society Copyright 2010, American Chemical Society Copyright 2003, Wiley.

By applying a charged jet, electrospinning processing can stretch the liquid into jet with small diameter under the action of surface tension, where the strong shearing action of the electric field induces more regular orientation arrangement (Li and Xia, 2004a; Xue et al., 2017). In addition, due to the fine diameter of the filaments, multi-axially oriented network structure can be further formed to prepare functional devices. As illustrated in Figures 3A–C), (Xue et al., 2019), liquid is squeezed out of the spinneret due to surface tension, creating pendant droplets. When energized, electrostatic repulsion deforms the droplet into a Taylor cone, from which a charged jet is ejected, and finally deposit on the grounded collector (Sun et al., 2014; Liao et al., 2018). Notably, various secondary structure can be constructed by tuning the electrospinning parameters (Figure 3D) (Yang et al., 2018). Especially, electrospun fibers with core-shell are produced *via* a coaxial conical jet under a high-voltage electrostatic field (Jiang et al., 2005). Li et al. (Li and Xia, 2004a) prepared fiber tubes by coaxial electrostatic spinning technology; Chen et al. (Chen et al., 2010) prepared core-shell structure microfiber of nanowires in microtubules by multi-fluid coaxial electrostatic spinning technology. Zhao et al. (Zhao et al., 2007) fabricated multi-channel fiber tubes by combining coaxial electrospinning technology with double-nozzle side-by-side electrospinning technology. This multi-cavity structure facilitates controlled release of drug in practical application (Wang et al., 2010; Cheng et al., 2019).

### Applications of Electrospinning in Bone Repairing and Artificial Blood Vessels

Electrospinning, as a preparation method of nanofibers, has been widely used in drug delivery (Li W. et al., 2015; Yu et al., 2015; Cleeton et al., 2019), and wound dressing (Ghosal et al., 2019; Séon-Lutz et al., 2019; Yue et al., 2021). Additionally, the rapid integration and simple fabrication of shaped fibers also enable electrospinning to be applied in the field of biomedical application such as bone repairing (Cheng et al., 2020; Kandasamy et al., 2020; Ma et al., 2021) and artificial blood vessels (Jang et al., 2020; Zhao et al., 2021).

**FIGURE 4:**
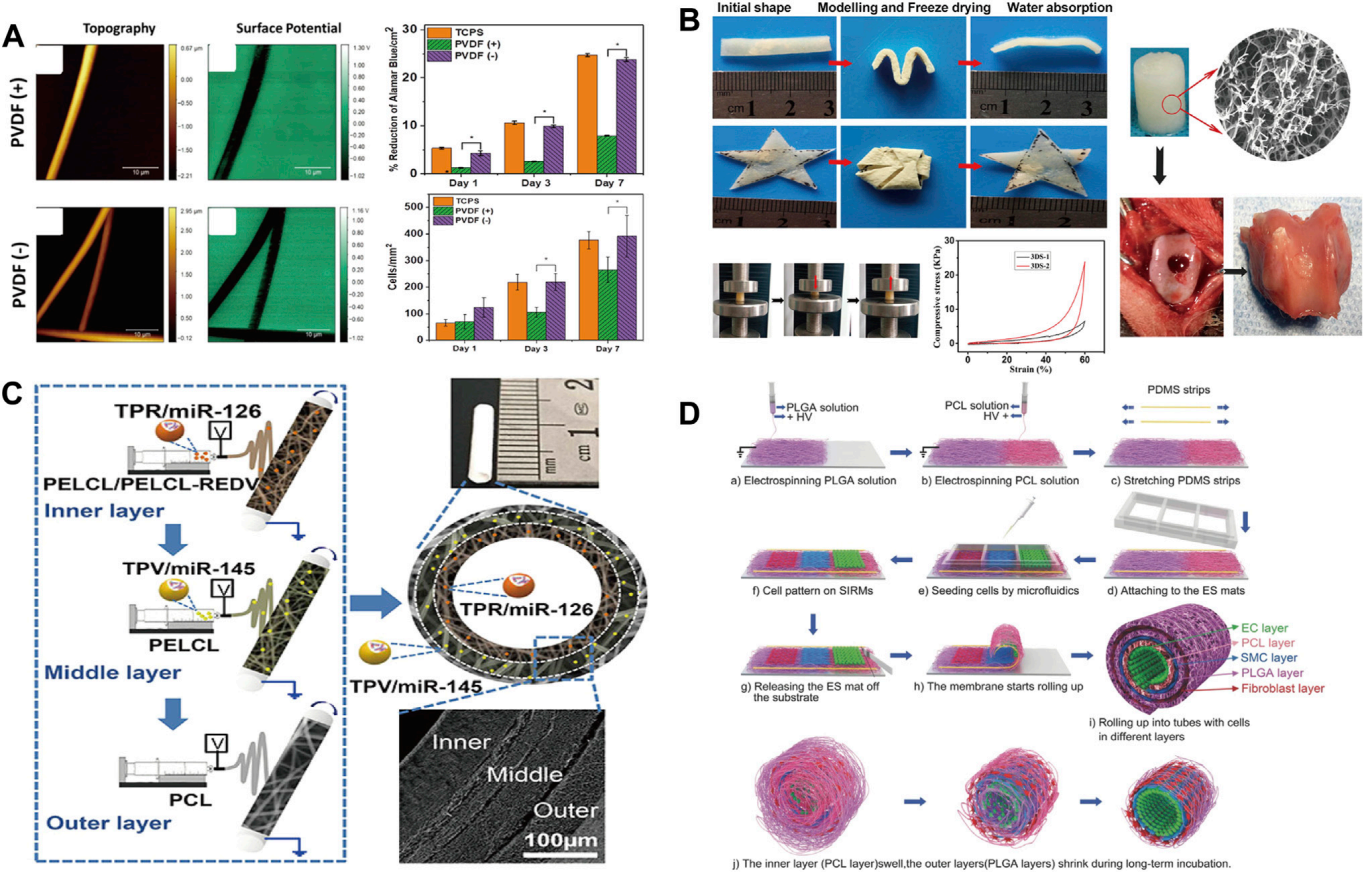
**(A)** Surface morphology and cell proliferation of electrospun PVDF fibers prepared at different voltages (Szewczyk et al., 2018) Copyright 2018, American Chemical Society; **(B)** Water absorption property. Photographs showing the scaffold shape recovery after water absorption and the mechanical properties and biocompatibility of samples (Chen et al., 2016) Copyright 2016, American Chemical Society; **(C)** Schematic diagram of multi-layer electrospinning and actual sample diagram (Wen et al., 2020) Copyright 2020, American Chemical Society; **(D)** Schematic illustration of fabrication of the cell-laden multilayered PCL–PLGA tubes (Cheng et al., 2017) Copyright 2017, Wiley.

Although bone grafts can be autografts or allografts, the source of the graft and the strong physical rejection forced researchers to look for more suitable substitutes to mimic the structure and function of bone tissue, while the enriched secondary structure and assembly geometry of electrospinning offer a promising solution (Jones and Hench, 2003). Ibrahim et al. (Yahia et al., 2019) prepared a PCL and chitosan/polyethylene oxide (CS/PEO) composite sandwich nanofiber by electrospinning. The unique sandwich porous three-dimensional structure can effectively promote the deposition of apatite, and the CS on the surface reduces the water contact angle of the material from 116.6 to 57.6, which improves the adhesion of cells. An interesting phenomenon is also observed by Stachewicz (Szewczyk et al., 2019) that by applying negative voltages, the fluorine content on the polyvinylidene fluoride (PVDF) surface is changed and effectively promote the cells on the electrospun fibers to proliferate more, which was favorable for cell adhesion, proliferation and differentiation (Figure 4A). Besides 2D nonwovens, electrospinning can also be used to fabricate 3D devices. Chen (Chen et al., 2016) prepared flexible porous 3D scaffolds by electrospinning, and the scaffolds exhibited excellent bone regeneration and elasticity (Figure 4B). The excellent cytocompatibility deposition ensure its subsequent application of bone repair in rabbits and simultaneously, as a flexible material, it can be applied to various environments and different parts of the body.

Cardiovascular disease, as a global high-mortality disease, has received extensive attention from the academic community (Goins et al., 2019). There is a great demand for artificial blood vessels in clinical operations. The conventional processing technology cannot process small-diameter blood vessels, and due to the existence of thrombosis (Wang et al., 2020b; Wang et al., 2020c) and intimal hyperplasia (Jeong et al., 2020; Park et al., 2020), the artificial blood vessels are difficult to keep unobstructed (Tang et al., 2018). At present, artificial blood vessels are mainly prepared by electrospinning (Murphy and Atala, 2014). Most importantly, the combination of materials and structure derived from electrospinning can endow artificial blood vessel with more tunable properties. Wen et al. (Wen et al., 2020) prepared artificial blood vessels with a three-layer structure by continuously spinning different electrospinning fabrics (Figure 4C). Through the combination of different materials and different cells in the human body, the blood vessels can be unobstructed and exhibits excellent anticoagulation and antiproliferative properties. Cheng et al. (Cheng et al., 2017) employed double nozzles to spin polycaprolactone (PCL) and poly (lactic-co-glycolic acid) (PLGA) fabrics and stack them by winding to form artificial blood vessels (Figure 4D). Due to the expandability of PCL and the shrinkage of PLGA, the rolled composite material perfectly balances the size transformation of PCL and PLGA, and maintains the shape stability inside the artificial blood vessel. Meanwhile, the relatively slow degradation rate of the inner PCL layer were beneficial for the durable mechanical support of the initial structure of the tube, while the faster degradation of the PLGA promoted cell growth and angiogenesis. Although the pore size of electrospun materials often prevents cell infiltration, the control of mass density and geometry as well as fiber orientation opens up new opportunities in the field of artificial blood vessels, leading to better artificial blood vessel grafts in the future (Niklason and Lawson, 2020).

## Micro Injection Molding

### Principle of Micro Injection Molding

**FIGURE 5:**
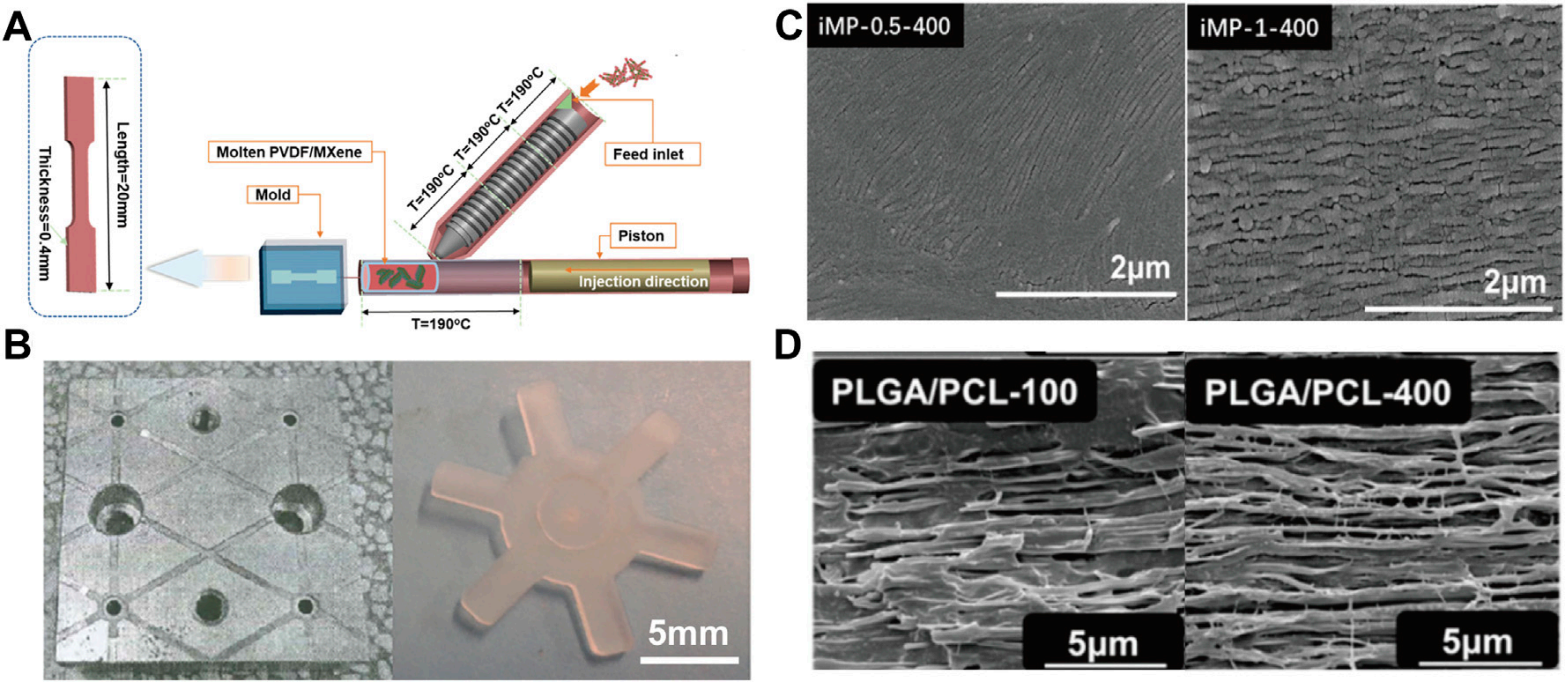
**(A)** Schematic diagram of the micro-injection device (Han et al., 2021) Copyright 2020, American Chemical Society; **(B)** Micro injection molded parts (Wang et al., 2019) Copyright 2019 Wiley; **(C)** SEM images of high shear induced crystallization (Han et al., 2021) Copyright 2021, American Chemical Society; **(D)** SEM images of PLGA/PCL materials prepared by micro-injection at different injection rates (Cai et al., 2021) Copyright 2021, Elsevier.

The micro-injection process is currently one of the most important technologies for preparing polymeric micro-device due to low manufacturing cost, high precision, short molding cycle and the ability to prepare complex parts (Figures 5A,B) (Wang, 2019; Piccolo et al., 2021). Although similar with traditional injection molding process, the melt in micro-injection can be described as an ideal case of laminar flow, the shear in the micro confined channel is significantly greater than that of the traditional injection molding process (Jiang et al., 2018). Accordingly, the ultrahigh shearing (10^7^ s^−1^) can lead to increased degree of orientation and crystallinity by shear-induced crystallization (Cai et al., 2021). For instance, shish-kebab structures are keen to form during micro-injection (Figure 5C) (Han et al., 2021) and thus realize enhancement of physical and chemical properties of the material (Shi et al., 2019). Special crystal modification such as the *β* crystal of PVDF can also be scalarly fabricated with the assistance of the ultrahigh shear in micro-injection. Nie (Guo et al., 2020) successfully prepared PVDF piezoelectric parts with a relative fraction of *β* crystals of 52% by micro-injection, and the open-circuit voltage density reached ∼11 V/cm^2^, with over 4-fold improvement as compared to that fabricated by conventional cast method. Except from crystal, the ultrahigh shear can also promote the mechanism of *in-situ* fibrillation. It is worth noting that the extremely high shear rate and fast cooling rate of micro-injection can also tailor special interlocking structure which cannot obtained in conventional *in-situ* fibrillation processing (Cai et al., 2021) (Figure 5D).

### Applications of Micro Injection Molding in Cell Proliferation and Bone Tissue Engineering

**FIGURE 6:**
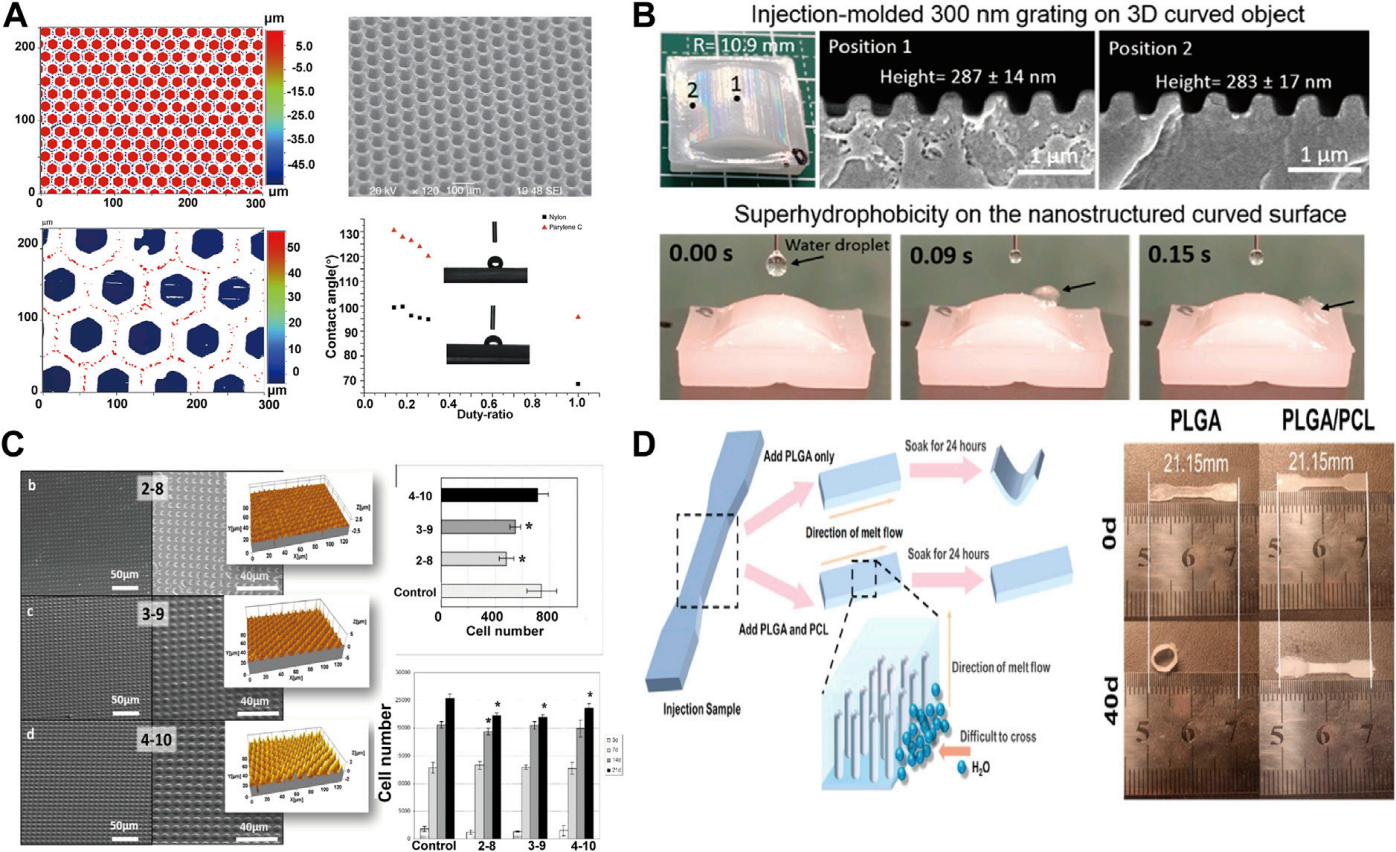
**(A)** Preparation of honeycomb nylon and water contact angle data (Wang et al., 2018) Copyright 2018 Springer Nature; **(B)** Macroscopic curved submicron superhydrophobic surface structures fabricated using micro injection molding and nanoimprinting (Lee et al., 2019) Copyright 2019, American Chemical Society; **(C)** SEM images and cell proliferation images of micro-injected samples with different pore sizes (Zanchetta et al., 2015) Copyright 2015, American Chemical Society; **(D)** Micro injection molding enhances dimensional stability of materials through *in-situ* fiberization (Cai et al., 2021) Copyright 2021, Elsevier.

In the field of cell proliferation, the proliferation and differentiation of cells can be effectively regulated by adjusting different processing parameters. The micro structured surfaces will affect the proliferation and differentiation of cells and are increasingly important for biological and clinical applications (Benoit et al., 2008; Yim et al., 2010). The micro-injection process can realize the rapid industrial preparation of microstructures. The super hydrophobic or super hydrophilic design of micro surfaces can be well used in medical devices, especially the local microenvironment (topography and strength) can decisively affect the ability of cells to self-renew and differentiate (Yatsushiro et al., 2016; Shiu et al., 2018). Wang et al. (Wang et al., 2018) fabricated honeycomb-like micro surface arrays with different contact angles by means of micro-injection (Figure 6A). Li et al. (Lee et al., 2019) successfully achieved the preparation of curved parts with sub-micron surface morphology through micro-injection and nano-imprinting (Figure 6B). Zanchetta et al. (Zanchetta et al., 2015) fabricated devices with different microscopic features and studied the proliferation and differentiation of cells under different microscopic features (Figure 6C). Differences in micro surfaces can cause cells to differentiate in different directions while the difference in pore size leads to the difference in proliferation efficiency. The micro injection molding process can prepare large-scale devices for cell proliferation and differentiation (Matschuk et al., 2010). Micro-injection has also shown great potential in the field of bone tissue engineering, to cooperate with new materials and achieve comprehensive biological and mechanical properties (Chen et al., 2017; Yang X. et al., 2021). Cai et al. (Cai et al., 2021) prepared PLGA/PCL fixation plate by *in-situ* fibrillation in micro-injection process (Figure 6D). The dimensional stability of the material is improved with improved mechanical properties, where the plate maintained its original size after being soaked in phosphate-buffered saline (PBS) for 40 days, laying a foundation for the subsequent preparation of bone repair materials.

## Inkjet Printing and Laser Assisted Bioprinting

### Principle of Inkjet Printing and Laser Assisted Bioprinting

3D printing allows rapid transformation of computer-aided designs into complex 3D printed parts, enabling rapid on-demand manufacturing (Lee et al., 2017; Wang et al., 2017). Compared with conventional processing, 3D printing technology has better customization and can prepare complex geometry for special purposes, which has great potential in the field of biomedical engineering. Especially, inkjet 3D printing (Murphy and Atala, 2014; Saunders and Derby, 2014; Knowlton et al., 2015; Mandrycky et al., 2016; Lee et al., 2019) and laser assisted bioprinting (Barron et al., 2004; Palla-Papavlu et al., 2011; Dou et al., 2021) exhibits rapid development and inspiring potential, which deserved an opportune review and attention.

**FIGURE 7:**
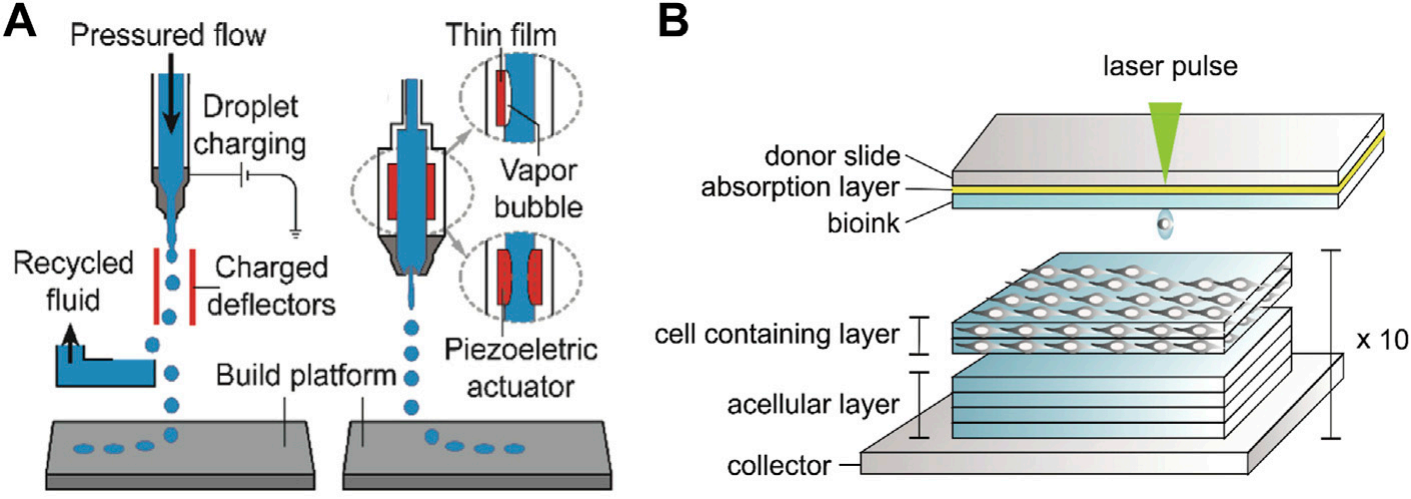
**(A)** Schematic diagram of the inkjet 3D printing device (Zhou et al., 2020) Copyright 2020, Wiley; **(B)** Schematic diagram of the laser-assisted bioprinting system and printing of the 3D stromal mimicking structures (Sorkio et al., 2018) Copyright 2018, Elsevier.

Inkjet printing is realized by selectively depositing droplets of printing material to form target entities (Mei et al., 2005; Zhou et al., 2020). The low-viscosity fluid namely the ink is ejected through the nozzle and moves with the platform to accurately deposit on the printing platform to solidify (Figure 7A). Printing inks suitable for biomedical engineering should have biocompatibility, biological function, printability and structural stability, while hydrogels (Boland et al., 2007; Nakamura et al., 2010; Kyle et al., 2017) featuring multifunctionality are similar to extracellular matrix and biological tissues, making it become an ideal candidate for bio ink and realizing the gel 3D printing (Zhang et al., 2020). Although Gel 3D printing is widely used in the fabrication of complex devices, the specific fluid properties and curable nature of droplets also hinder the development of inkjet printing (Zhang B. et al., 2018). The functionality of printed parts depends primarily on the inherent bioactivity of the hydrogel and the retention of contained cells and growth factors (Unal and West, 2020; Advincula et al., 2021). However, the contradiction between printability and structural stability of most hydrogels is not conducive to obtaining high printing accuracy, self-support, and structural fidelity, which is an urgent problem to be solved in the field of bio-3D printing.

The laser assisted bioprinting (LAB) printer consisting of a laser generator, a laser path adjustment module and a cell transfer module can focus laser pulse to the laser absorbing layer in the cell transfer module and thus generate air bubbles to push the bioink (usually the sol embedded in cells) downward to print patterns with high cell viability (Figure 7B) (Duocastella et al., 2009; Serra et al., 2009; Duocastella et al., 2010; Guillemot et al., 2010; Unger et al., 2011; Sorkio et al., 2018). Compared with traditional printing methods, LAB achieves higher printing accuracy and resolution through laser deposition (Guillotin et al., 2010; Cheptsov et al., 2019). The emergence of LAB enables 3D printing of structural materials with high cell density (Ringeisen et al., 2010; Kérourédan et al., 2019), and further guarantees the combination with other printing technologies to realize the preparation of various human tissues and organs. It is worth noting that the productivity and efficiency of LAB is not competitive with other printing methods due to the small amount of transferable biological material in each laser pulse, which limits the development of LAB printing technology.

### Applications of Inkjet Printing and Laser Assisted Bioprinting in Tissue Engineering

**FIGURE 8:**
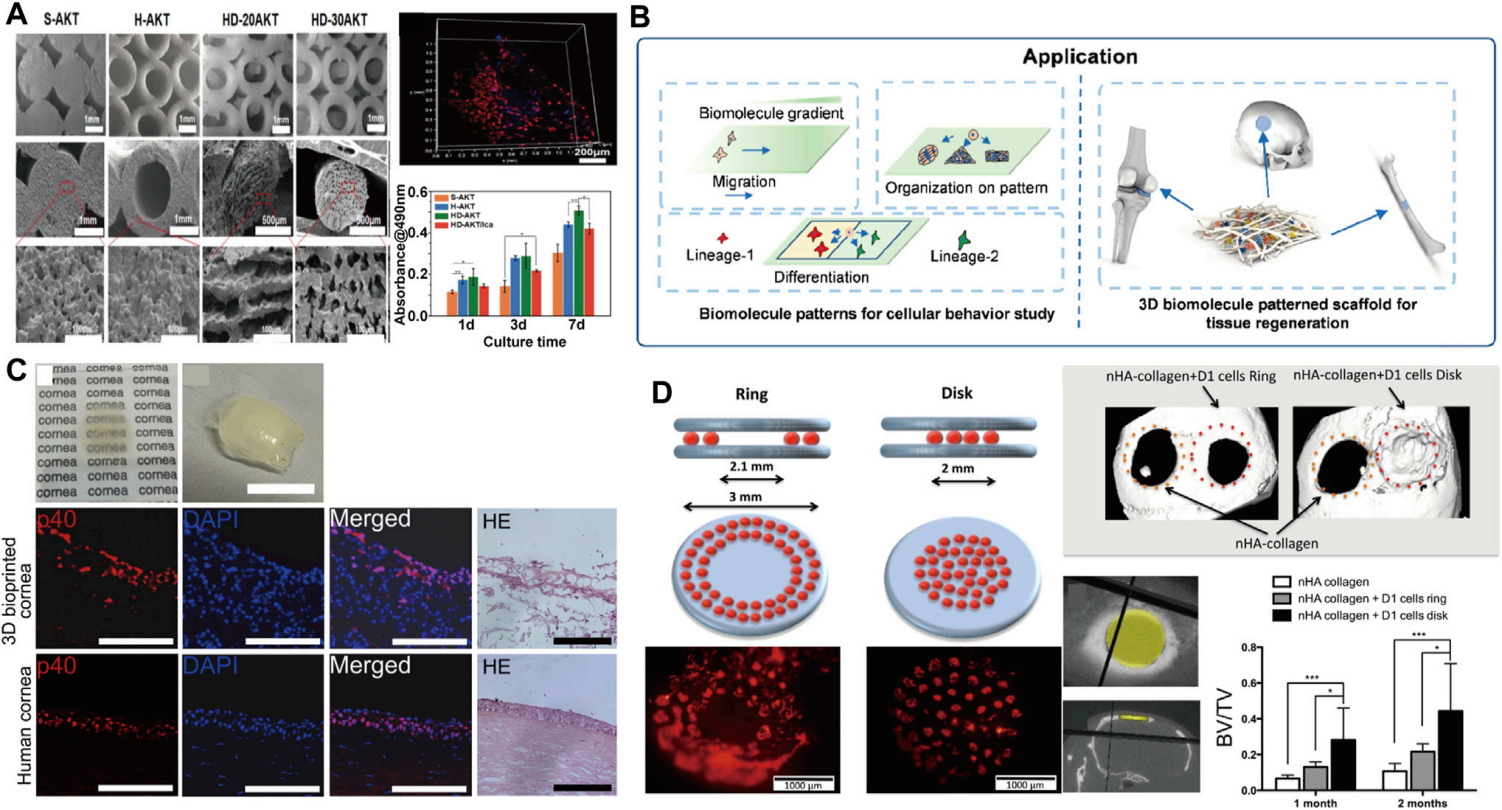
**(A)** SEM topography and cell proliferation data of 3D printed fibers with different pore sizes (Li Y. et al., 2019) Copyright 2019, Wiley; **(B)** With the printing, biomolecule patterns were applied in cellular behavior study and tissue regeneration (Li et al., 2020) Copyright 2020 American Chemical Society; **(C)** 3D cornea from hESC-LESCs and Human adipose-derived mesenchymal stem cells (hASCs) fabricated using laser-assisted bioprinting (Sorkio et al., 2018) Copyright 2018, Elsevier; **(D)** Schematic representation of the *in vivo* laser assisted bioprinting geometries tested and the experimental diagram of mice calvaria restoration (Keriquel et al., 2017) Copyright 2017 Springer Nature.

The ability of 3D printing technology to prepare complex structures is widely used in the field of biomedical engineering, (Gao et al., 2018), because 3D printing can effectively modulates the morphological structure of parts (Ma et al., 2018; Wang et al., 2021). For instance, the ability of cells to proliferate and differentiate depends on the morphological structure of the component itself. Ajdary (Ajdary et al., 2019) prepared nanocellulose structural parts with different porosity by direct ink writing (DIW) printing. The porous structure and interconnected network structure increase the permeability of cells in the structure, which facilitates the transport of nutrients and the discharge of metabolic wastes, and leads to an increase in the ability of cells to proliferate. Li et al. (Li T. et al., 2019) also controlled cell proliferation by preparing hollow tubes with different inner diameters by DIW (Figure 8A), where the high specific surface area and surface adhesion of hollow tubes resulted in enhanced cell proliferation and promoted expression of osteogenic genes in cells. Moreover, the 3D printing can endow medical device with shape memory effect which offer new methods in clinical application. In addition, the use of biomolecules for inkjet printing can further modulate cell behavior, with the hope that it can mimic natural tissue and organ growth (Figure 8B) (Li et al., 2020). Nowadays, this method has been widely used in bone and tissue engineering.

Compared with traditional 3D printing technology, LAB technology not only increases the proportion of biological tissues, but also avoids the cell death during processing, setting off a new storm in biomedical engineering. Dias et al. (Dias et al., 2014) studied LAB printing of embryonic stem cells, by which embryoid bodies can be formed. In addition to the development of LAB in the printability of stem cells, LAB has also made achievements in the fields of tissue engineering and bone repair. Sorkio et al. (Sorkio et al., 2018) successfully prepared clinically applicable corneal structures by combining human embryonic stem cell-derived limbal epithelial cells (hESC-LESCs) with collagen I for LAB printing (Figure 8C). Keriquel (Keriquel et al., 2017) induced bone repair by depositing hydroxyapatite particles into critical-sized calvaria defects in mice (Figure 8D). All these works expand the application of LAB technology, and provide more options for clinical surgery to avoid secondary surgery. It is worth noting that the high cost and relatively small geometry figure limit the development of LAB technology and at the same time, the parameters of LAB printing technology are not fully clarified. Consequently LAB is still relatively immature for the fabrication of 3D tissue structures, which also provides a new direction for 3D printing.

## Summaries and Perspective

Advanced machining technologies continue to evolve with their unique functionality and manufacturing speeds. Although different processing methods can obtain the same medical device, due to the different force field and temperature field during the processing, its morphological and structural properties also have advantages and disadvantages. This review summarizes the application of advanced processing technology in new medical materials and new devices in a timely manner (Table 1). It is not difficult to imagine that there will be more breakthroughs in advanced processing technology on this basis, but there are still some challenges.

**TABLE 1:** Application and features of various processing techniques in bone and tissue engineering.

Processing techniques	Stress and temperature features	Material	Application in cell proliferation	Application in bone repair	Application in artificial blood vessels
Micro injection molding	Ultrahigh shear and cooling rate	Thermoplastic polymer	Microarrays	• Improved mechanical properties	
				• Dimensional stability	
Electrospinning	• Strong shearing	Polymer solution (requiring coagulation bath)	• Heteromorphic materials	Flexible devices enhance application range	• Dimensional stability
					• Multilayer
	• Secondary assembly structure		• Voltages regulating cell proliferation		
Rotation extrusion	Multi-dimensional controllable flow	Thermoplastic polymer		Enhanced axial strength and resistance to radial deformation	
3D printing	• Customizability	• Bioink	3D structures regulating cell proliferation	Controlled functionality to promote bone repair	
	• High precision	• Hydrogel; cell			
	• Bioactive	• Specific polymer droplets			

There are also issues to be addressed in the future development of advanced polymer processing. For equipment, iterative updates of processing equipment are required. Advanced processing equipment must be compatible with scale and integration in order to amplify its advantages. This places stringent requirements on manufacturing equipment, which must be highly intelligent and suitable for continuous production. In terms of materials, large-scale preparation technology of materials suitable for advanced processing technology is also highly urgent. The range of materials available for processing technologies such as electrospinning or 3D printing is relatively narrow, because of demanded processing parameters. Therefore, how to expand the material universality of these processing technologies has also become a current research hotspot.

Through advanced processing technology, functional medical materials and devices endow modern medicine with unimaginable convenience. Especially with the development of information technology, it also endows these technologies with the possibility of digital manufacturing and great potential with digital twin technology. These emerging processing technologies will greatly expand the feasibility and diversity of medical technologies, thereby revolutionizing the traditional medical industry.
